# Supplementary material for: Rhodopsin-cyclases for photocontrol of cGMP/cAMP and 2.3 Å structure of the adenylyl cyclase domain
Source: Nat Commun. 2018 May 24;9:2046. doi: 10.1038/s41467-018-04428-w (PMC5967339; doi:10.1038/s41467-018-04428-w)
Supplement: Supplementary file 1 — Supplementary Information [file 41467_2018_4428_MOESM1_ESM.docx]

Supplementary Material for

**Rhodopsin-cyclases for photocontrol of cGMP/cAMP and 2.3 Å structure of the adenylyl cyclase domain**

Scheib et al.

**This PDF file includes:**

Supplementary Figures 1-15

Supplementary Tables 1-4


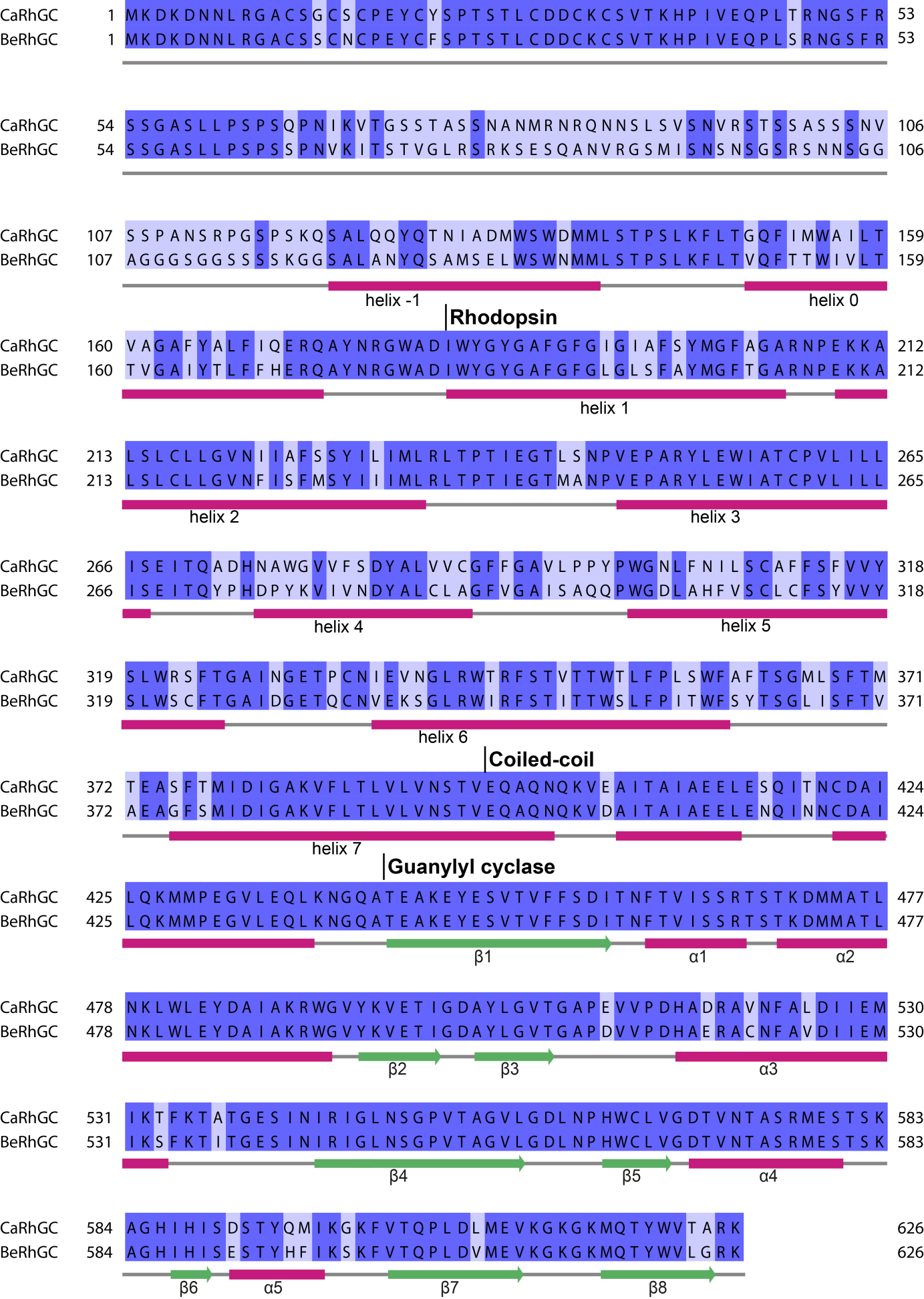


**Supplementary Figure 1 | Comparison of CaRhGC and BeRhGC amino acid sequences**. The pairwise sequence alignment (Emboss Needle 6.6.0, gap penalty 15) is based on RhGC from *Catenaria anguillulae* (CaRhGC, gb: MF939579) and RhGC from *Blastocladiella emersonii* (BeRhGC**,** gb: AIC07007.1). Identical amino acids are shaded dark blue, differences are shaded light blue. Both RhGCs are composed of three consecutive domains, the rhodopsin, the coiled-coil and the guanylyl cyclase domain (domain starts according to Avelar et al.^1^. Secondary structure elements (helices in magenta, β-strands in green) are depicted below the sequence alignement. For residiues 1 - 442 helix prediction is based on JPred4^2^ according to^3^, secondary structure elements within the guanylyl cyclase are based on the CaAC structure.


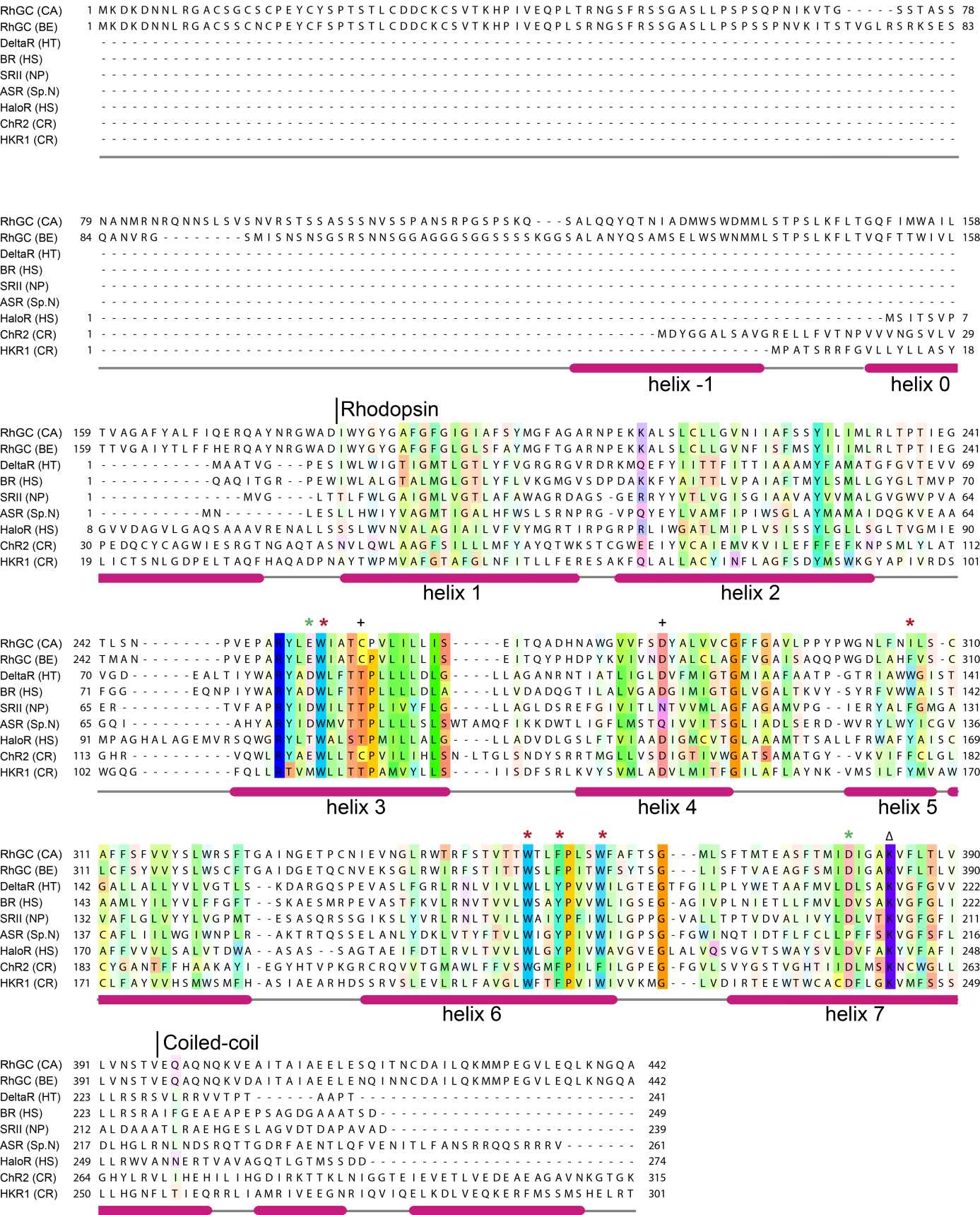


**Supplementary Figure 2 | Sequence alignment of rhodopsin domains from several species.** Sequences were aligned with the help of ClustalO^4^. Predicted helices (magenta, aa 1 - 442) based on JPred4^2^ are shown beneath the sequences and the predicted start of the rhodopsin and coiled-coil domains are indicated. Included are: rhodopsin domains from the rhodopsin guanylyl cyclases of *Catenaria anguillulae* RhGC (CA) (gb: MF939579), *Blastocladiella emersonii* RhGC (BE) (gb: AIC07007.1); delta rhodopsin: DeltaR (HT) of Haloterrigena *thermotolerans* (Uniprot: I4DST7); bacteriorhodopsin: BR(HS) of *Halobacterium salinarum* (gb: 10580964); sensory rhodopsin II: SRII (NP) of *Natronomonas pharaonis* (gb: 510870); anabena sensory rhodopsin: Anabaena SR (Sp.N) of *Nostoc* sp. PCC7120 (gb: 17132260); halorhodopsin: HaloR (HR) of *Halobacterium salinarium* (gb: 169235207); channelrhodopsin: ChR2 (CS) of *Chlamydomonas reinhardtii* (gb:158280944); and histidinkinase rhodopsin : HKR1 (CR) of *Chlamydomonas reinhardtii* (gi: 410699688). Symbols indicate key residues conserved among rhodopins: aromatic amino acids surounding the retinal chromophore are indicated by red stars. Lys^384^ (black triangle) in transmembrane helix 7 (TM7) is expected to bind the retinal chromophore covalently. Glu^254^ and Asp^380^ (green asterisks) may serve as counter-ions for the retinal Schiff Base. The “DC-pair residues” (Cys^259^ and Asp^283^ in RhGCs), which are important for photocycle kinetics in channel rhodopsins, are indicated by a black “plus”.

**
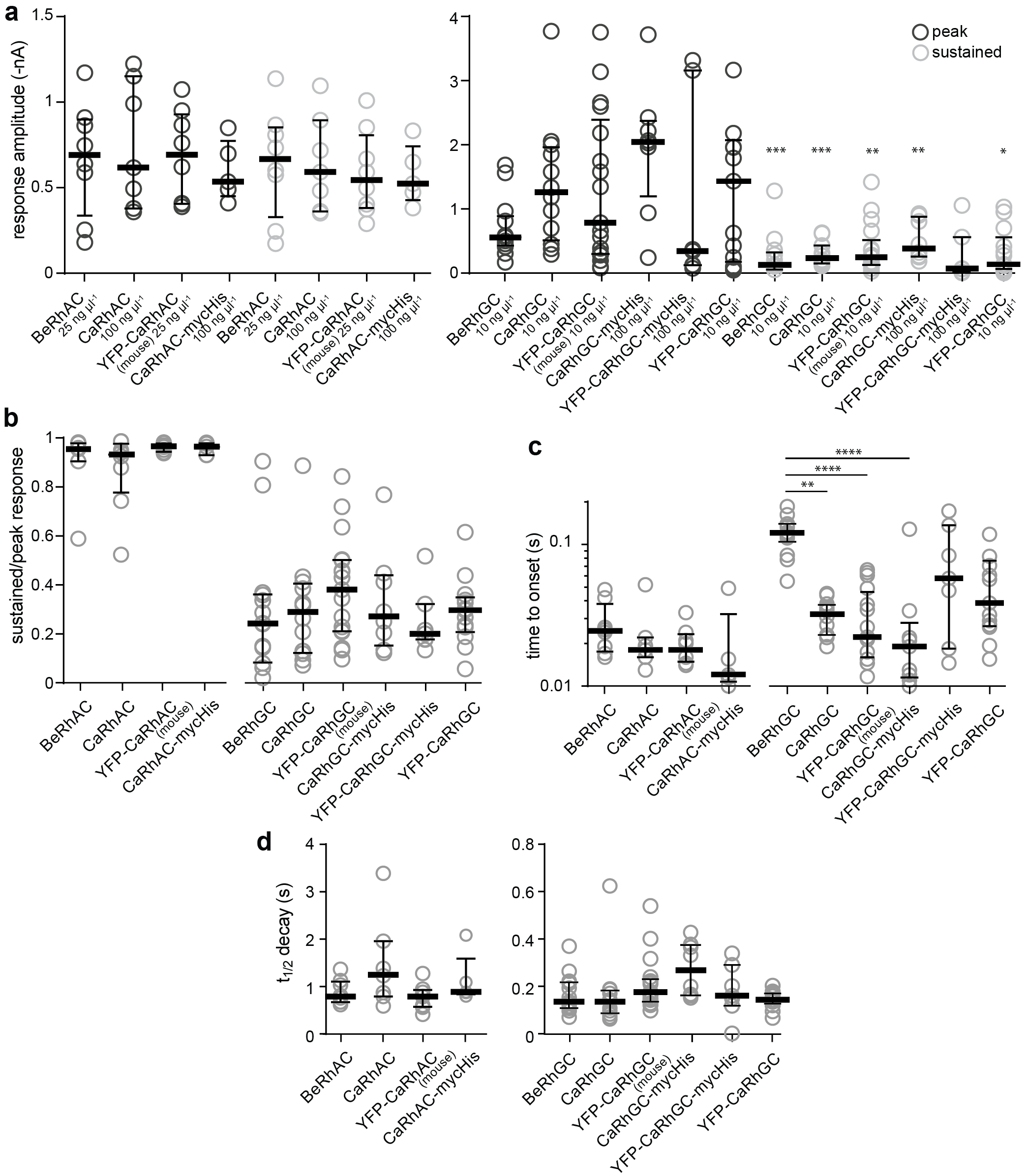
**

**Supplementary Figure 3 | Functional characteristics of the light evoked responses of RhAC and RhGC variants expressed in hippocampal neurons.** (**a**) Comparison between the transient peak response versus the sustained response during light stimulation; *** p = 0.0006, *** p = 0.0001, ** p = 0.0095, ** p = 0.007, * p = 0.025, Mann-Whitney test. Numbers next to the construct name indicate the concentration of rhodopsin DNA electroporated (ng µl^-1^); channel DNA was always 25 ng µl^-1^, mtSapphire 5 ng µl^-1^ (for panels b-d the same DNA concentrations as in subpanel a were used). (**b**) Ratio between the sustained response and the maximum peak recorded. (**c**) Time to onset of the response evoked by light stimulation of hippocampal neurons expressing RhAC and RhGC variants; ** p = 0.0029, **** p < 0.0001, Kruskal-Wallis test. (**d**) Time necessary for the response to decay to ½ of the value of the steady state response, after the stimulus ended; In all conditions RhAC variants were coexpressed with CNG(cAMP) and mtSapphire and RhGCs with CNG(cGMP) and mtSapphire. The light stimulation used was a 2 second pulse of 530 nm light at 27.3 mW mm^-2^ (except BeRhAC condition where the light intensity used was 19.2 mW mm^-2^). Bars show median and interquartile range.


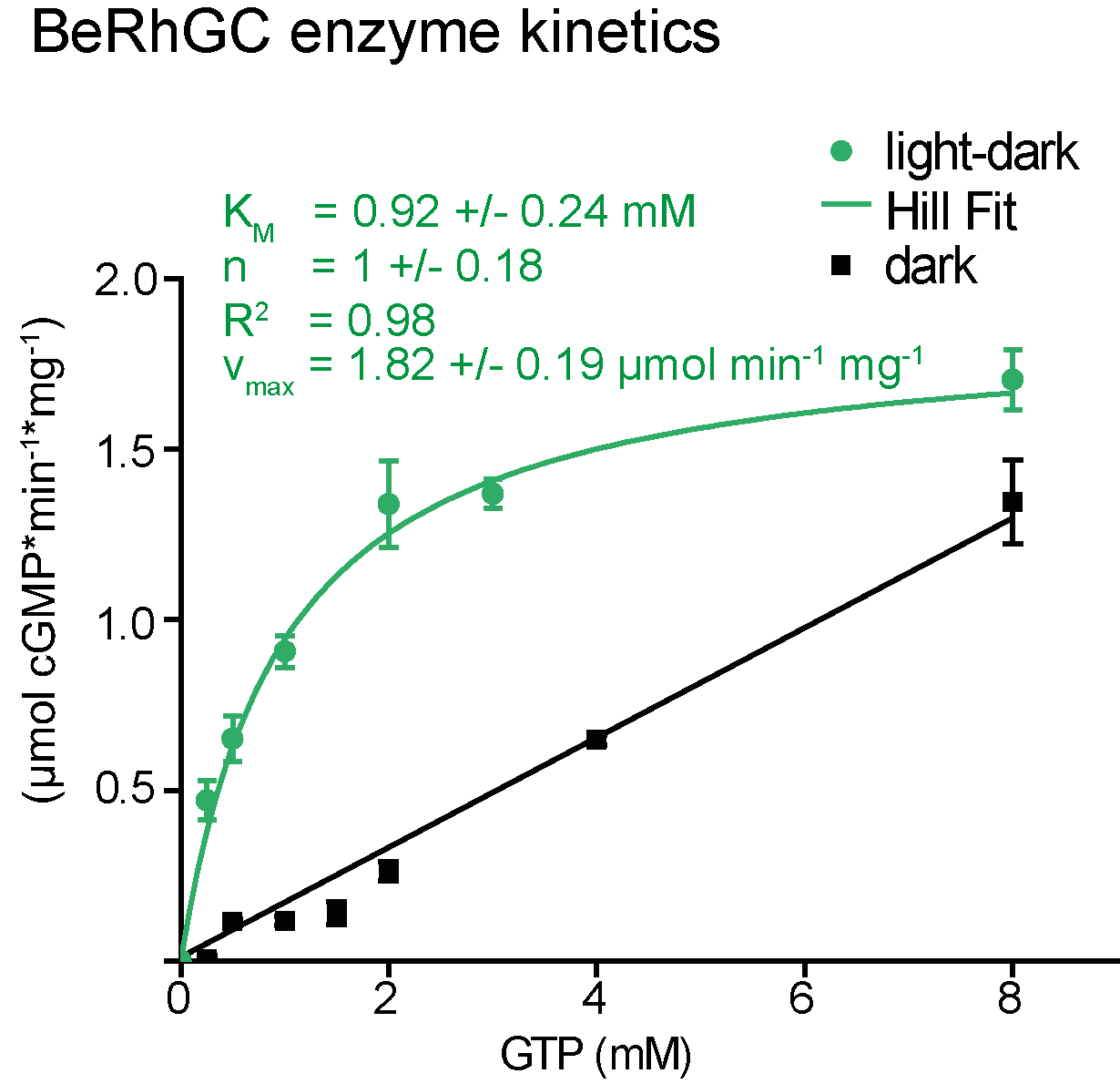


**Supplementary Figure 4 | Enzymatic activity of detergent purified BeRhGC.** BeRhGC was incubated with increasing concentrations of GTP/Mn^2+^ (0.25 – 8 mM, pH = 7.5, n = 3), samples were illuminated (light, green circles (dark activity subtracted) or kept in darkness (dark, black squares) for different time intervals. cGMP was quantified via RP-HPLC. The velocities of illuminated samples (dark activity subtracted) followed Michaelis-Menten kinetics and a Hill fit allowed to determine the enzymatic parameters, included in Table 1. The dark activity of BeRhGC increased linearly with the substrate concentration.


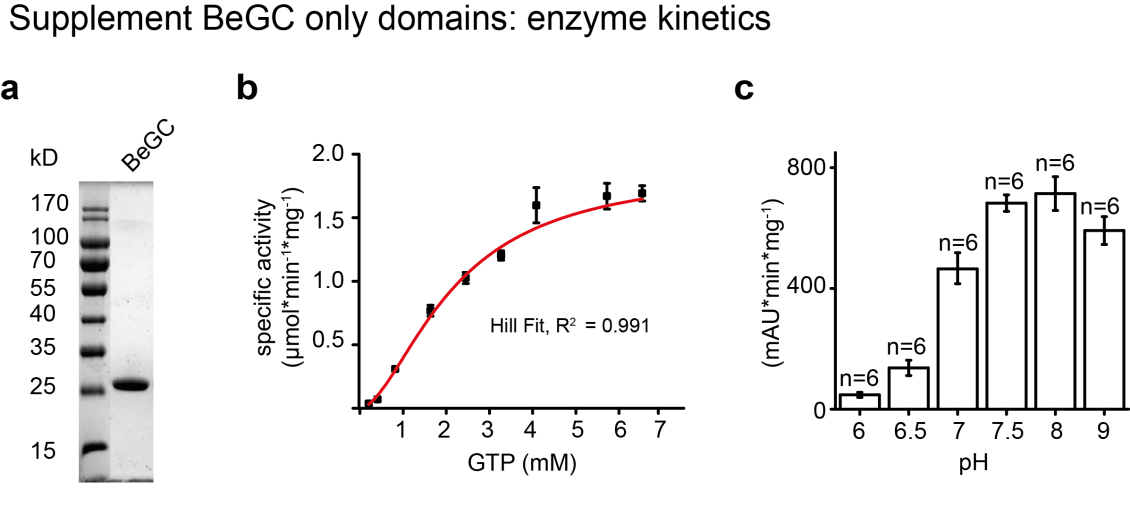


**Supplementary Figure 5 | Enzymatic activity of the constitutively active isolated guanylyl cylclase domain from *Blastocladiella emersonii* (BeGC).** (**a**) SDS-gel of purified BeGC (MW = 21.5 kDa). (**b**) Specific activity of BeGC, determined at increasing GTP concentrations (0.5 - 7 mM GTP/Mn^2+^, pH 7.5, n = 3). cGMP was quantified via RP-HPLC. Data was fitted according to Hill and the enzymatic parameters were determined, (K_M_ = 2.16 ± 0.30 mM, v_max_ = 1.89 ± 0.18 µmol min^-1^ mg^-1^, n = 1.72 ± 0.12) (see Table 1). (**c**) BeGC showed the highest activity at pH 8. cGMP peak areas (mAU*min) per mg protein were quantified after incubation of BeGC for 5 min in the presence of 1 mM GTP/Mn^2+^ at the indicated pHs. Bar graphs show data as means ± s.e.m.


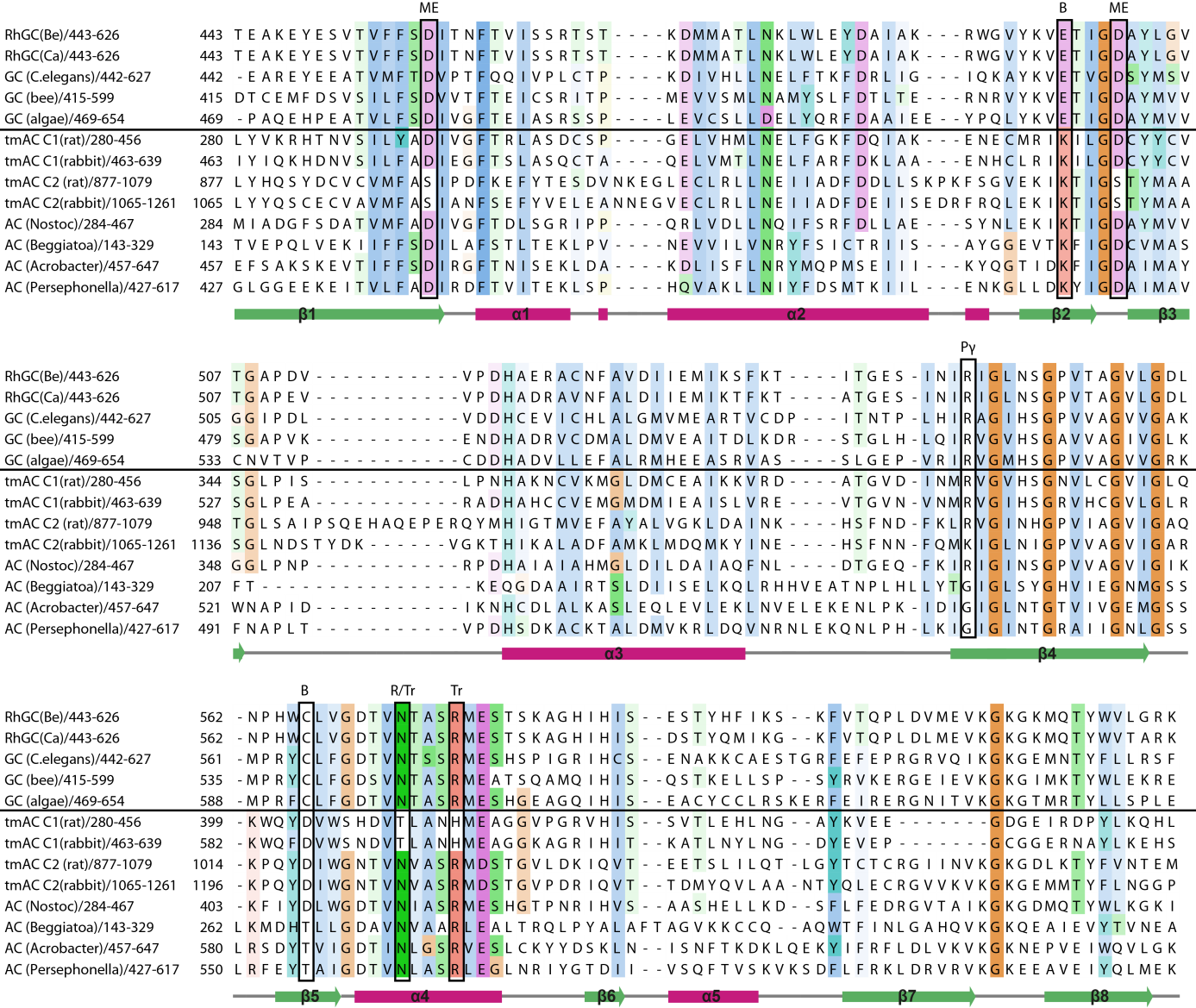


**Supplementary Figure 6 | Alignment of type III guanylyl- and adenylyl cyclases.** Different guanylyl and adenylyl cyclase sequences were aligned with the help of ClustalO^4^, coloring confers to 30 % conservation. Secondary structure elements are depicted below the sequence (helices in magenta, ß-strands in green) based on the CaAC crystal structure. For type III cyclases, seven key residues^5^ are mainly involved in base binding and catalysis. Among adenylyl cyclases the adenine base is bound by a Thr/Asp, Lys pair, while the guanine base within guanylyl cyclases is coordinated via a Cys, Glu pair (residues important for base binding are labeled with “B”). Two crucial aspartates (labeled with “ME”) coordinate two divalent ions (Ca^2+^/Mg^2+^/Mn^2+^), which are important for Pβƴ binding and are supposed to prime the ribose-3'OH for its nucleophilic attack on Pα. Residues involved in the stabilization of the transition state are highlighted with “Tr”. Residues involved in ribose binding and phosphate binding are labeled with R and Pƴ, respectively. The following sequences served as alignment input: guanylyl cyclase domain of RhGC from *Blastocladiella emersonii* (Be) (AIC07007.1), of RhGC from *Catenaria anguillulae* (Ca) (gb: MF939579), of *Caenorhabditis elegans* (C.elegans)(NP_510557), of *Apis mellifera* (bee) (NP_001012645), of *Chlamydomonas reinhardtii* (algae) XP_001700847.1. The adenylyl cyclase domain originates from type II transmembrane adenylyl cyclase of *Rattus norvegicus* (rat) (P26769.1), from the type V transmembrane rabbit adenylyl cyclase (rabbit) (CAA82562.1), from *Nostoc sp.* PCC7120 (Nostoc) (WP_010994837.1), from the marine bacterium *Beggiatoa alba* (ADC33127.1), from *Arcobacter butzleri* RM4018 (Acrobacter) (WP_012012652.1), and from *Persephonella marina* (Persephonella) (WP_015898934.1).


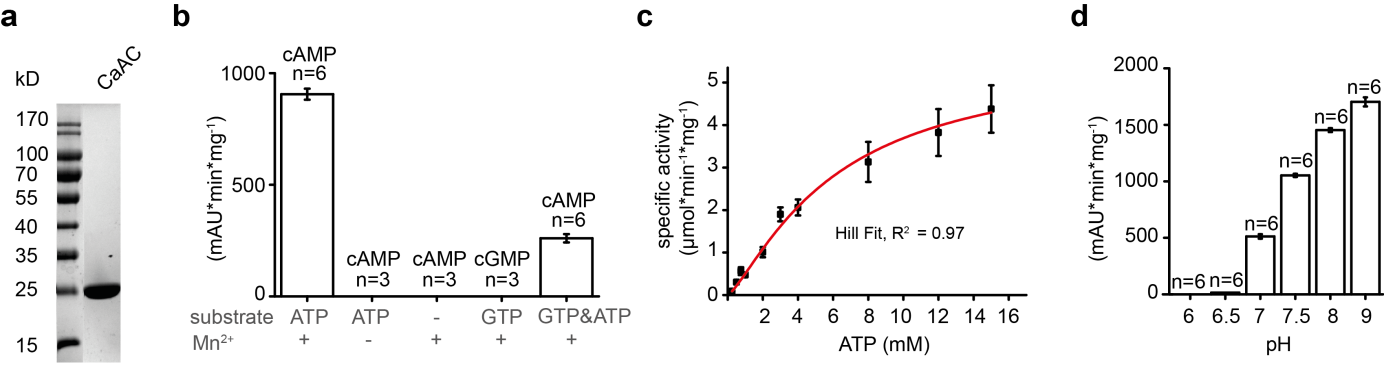


**Supplementary Figure 7 | Enzymatic characterization of the double mutated cyclase domain CaAC (E497K, C566D).** (**a**) SDS-gel of the purified CaAC (MW = 21.5 kDa). (**b**) Substrate and divalent ion dependence of enzymatic activity of CaAC. cNMP peak areas (mAU*min) per mg protein were quantified by RP-HPLC after incubation of CaAC for 5 min in the presence of ATP/GTP/Mn^2+^ (1 mM, pH = 7.5). (**c**) Specific activity of CaAC, incubated with increasing concentrations of ATP/Mn^2+^(0 -15 mM, pH = 7.5, n = 3). A Hill fit allowed the determination of the enzymatic parameters, included in Table 1. (**d**) CaAC showed the highest activity at pH 9. cAMP peak areas (mAU*min) per mg protein were quantified after incubation of CaAC for 5 min in the presence of 1 mM ATP/Mn^2+^ at the indicated pHs. Bar graphs show data as means ± s.e.m.

**
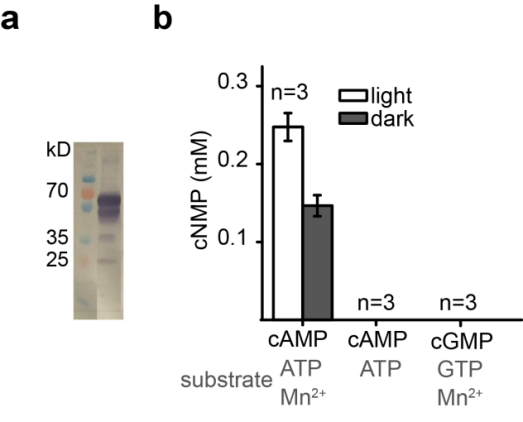
**

**Supplementary Figure 8 | Enzymatic characterization of detergent purified CaRhAC (E497K/C566D).** (**a**) Western-blot (anti-His) of detergent purified CaRhAC (MW = 70.6 kDa). (**b**) Enzymatic activity of detergent purified full-length CaRhAC in the presence of 1 mM ATP/GTP/Mn^2+^, 2 min illumination (522 nm, 1 µW mm^-2^) or darkness (pH = 7.5). Produced cNMP was quantified with the help of RP-HPLC.


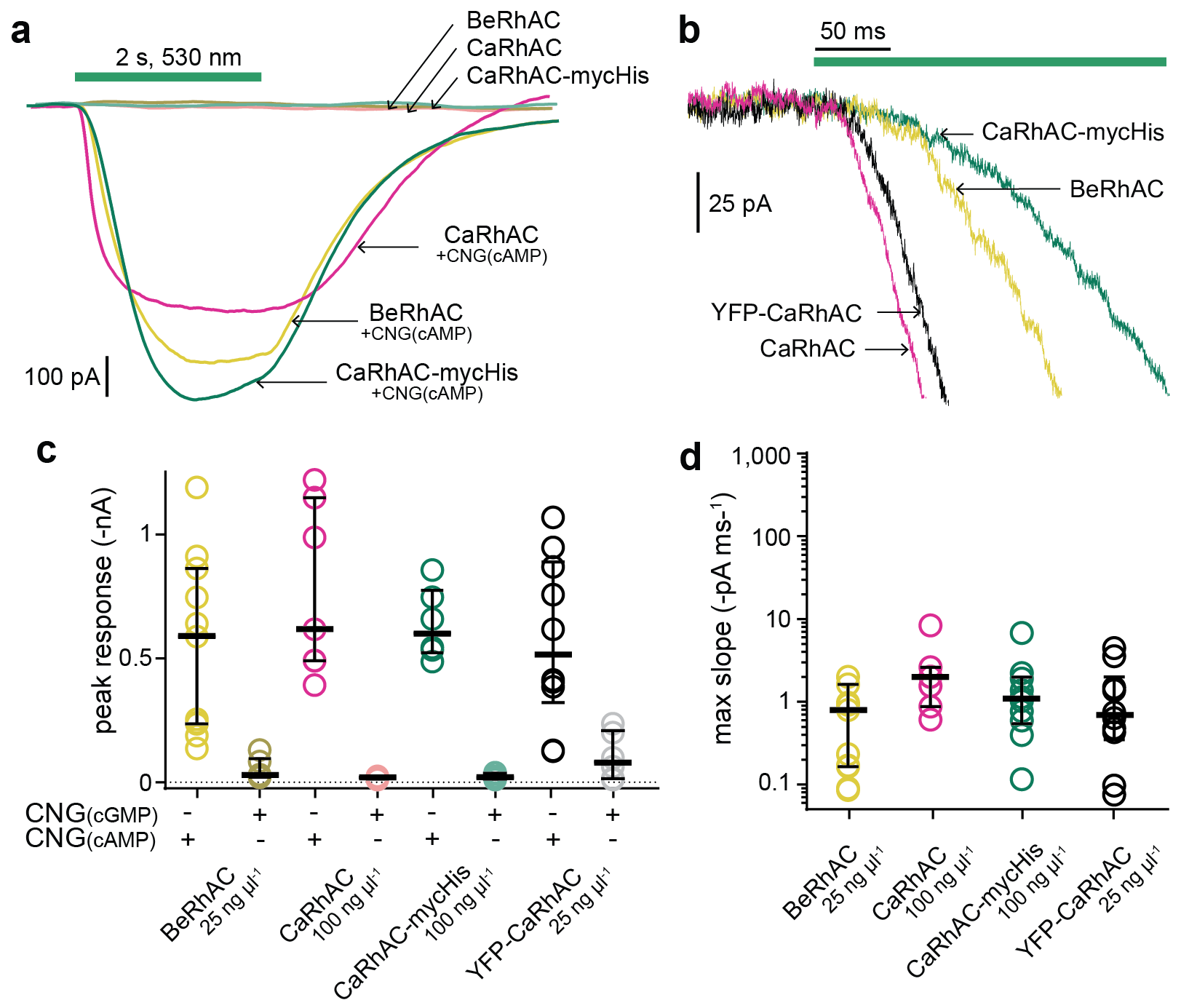


**Supplementary Figure 9 | Comparison of evoked responses of adenylyl cyclases derived from RhGCs in hippocampal neurons.** (**a**) Sample responses to 2 s green light (530 nm, 27.3 (CaRhAC, CaRhAC-mycHis) / 19.2 (BeRhAC) mW mm^-2^ of hippocampal neurons expressing the adenylyl cyclases together with CNG(cAMP) and CNG(cGMP) channels (both channels 25 ng µl^-1^, BeRhAC 25 ng µl^-1^, CaRhAC 100 ng µl^-1^, CaRhAC-mycHis 100 ng µl^-1^ electroporation DNA). (**b**) The first 200 ms of the evoked responses shown in (a) in comparison to the response of YFP-CaRhAC. DNA encoding for RhACs were coelectroporated with the DNA encoding cAMP sensitive CNG channel. (**c-d**) Comparison of maximum peak (c) and slope (d) of currents evoked by green light (530 nm, 2 s, ≤ 27.3 mW mm^-2^) in hippocampal neurons expressing un-tagged, c-terminal myc-His tagged and n-terminal YFP tagged RhACs; numbers next to the construct name indicate the concentration of rhodopsin DNA electroporated (ng µl^-1^). Channel DNA was always 25 ng µl^-1^, mtSapphire 5 ng µl^-1^. Note that comparable currents were evoked in neurons expressing rhodopsins with or without a c-terminal myc-His tag. Addition of an n-terminal YFP improved expression or surface localization of CaRhAC as comparable currents were obtain after electroporating 75% less DNA. n's = 11, 6, 7, 5, 6, 9, 10, 6, 11, 7, 10, 10 left to right. Bars show median and interquartile range.

**
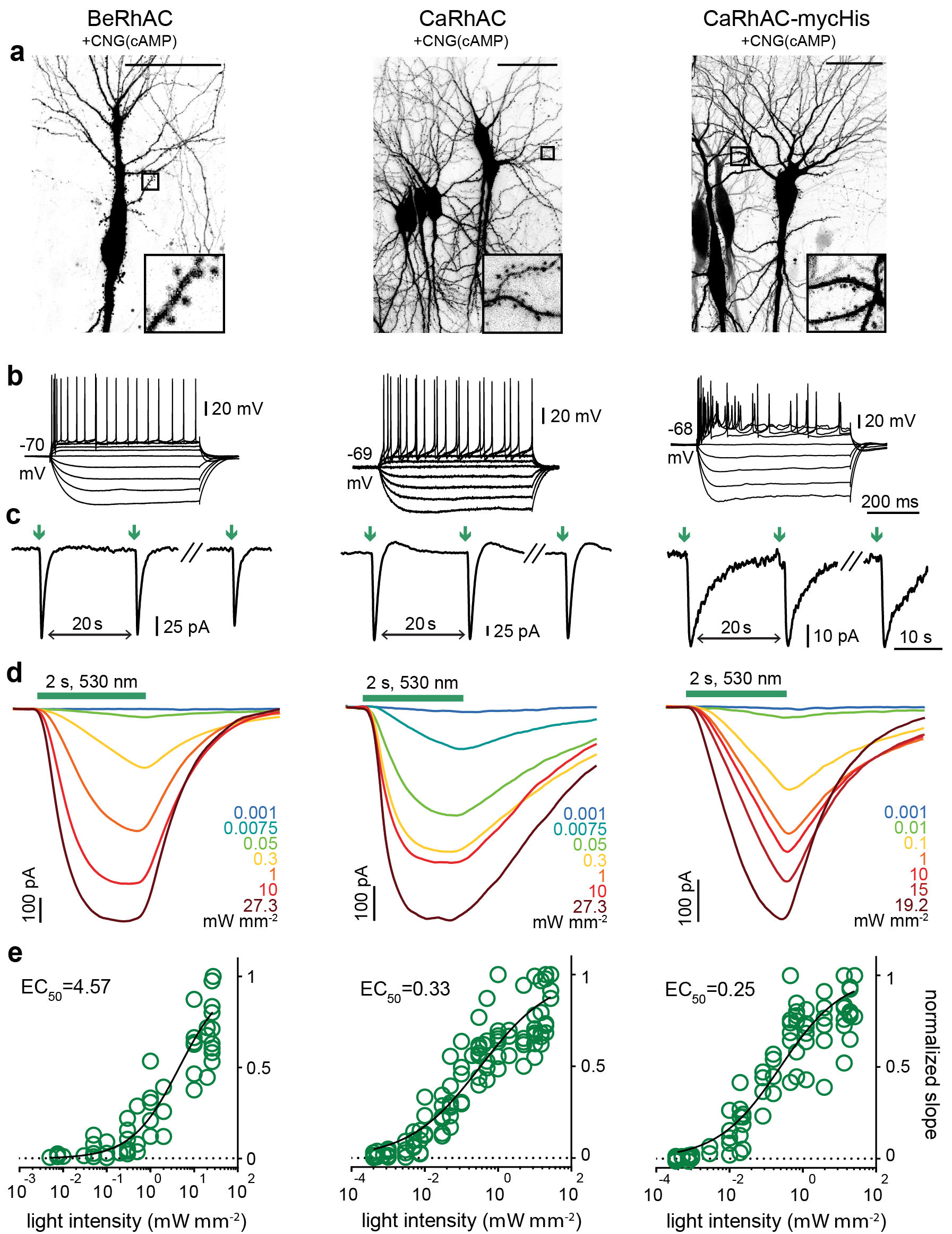
**

**Supplementary Figure 10 | Characterization of adenylyl cyclases derived from RhGCs in hippocampal neurons.** (**a**) Two photon images of mtSapphire fluorescence (excitation 800 nm) of hippocampal neurons 6-8 days after electroporation with: 25 ng µl^-1^ DNA encoding BeRhAC (left)**,** 100 ng µl^-1^ DNA encoding CaRhAC (middle)**,** 100 ng µl^-1^ DNA encoding CaRhAC-mycHis (right)**,** plus 25 ng µl^-1^ DNA encoding CNG(cAMP) and 5 ng µl^-1^ DNA encoding mtSapphire, scale bar 10 µm. Subscripts in panels b to e refer to the same mixtures of DNA as in a. (**b**) Whole-cell responses of hippocampal neurons (as above) to current injections of -400 pA to 400 pA in 100 pA steps. **(c)** Photocurrents evoked by 100 ms flashes of green light (530 nm) at 20 s intervals. Intensity (left to right): 27.3 mW mm^-2^, 27.3 mW mm^-2^, 0.019 mW mm^-2^. (**d**) Representative currents recorded in response to 2 s green light flashes of different intensity. (**e**) Light intensity-response relationship for ACs fitted with a quadratic equation. n's = 8**,** 7, 6 (left to right)**.**


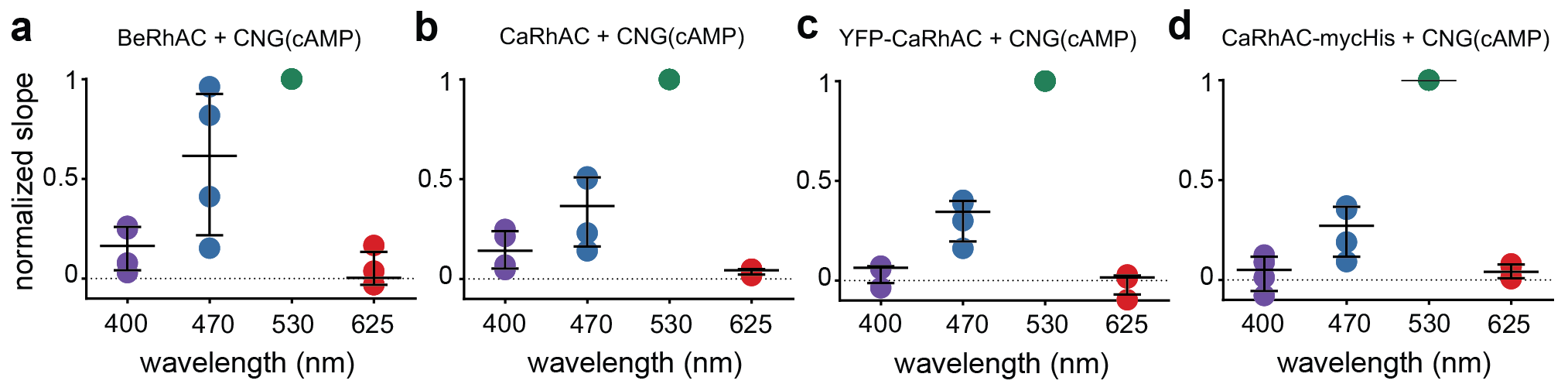


**Supplementary Figure 11 | Wavelength dependence of rhodopsin adenylyl cyclases in neurons.** Light-evoked currents from hippocampal pyramidal neurons expressing CNG(cAMP) channels together with the indicated rhodopsin at approximately EC_50._ **a**) BeRhAC 1mW mm^-2^ **b**) CaRhAC 0.03 mW mm^-2^,**c**) YFP-CaRhAC 0.05 mW mm^-2^, **d**) CaRhAC-mycHis 0.019 mW mm^-2^ n = 4, 4, 4, 4. Bars show median and interquartile range.


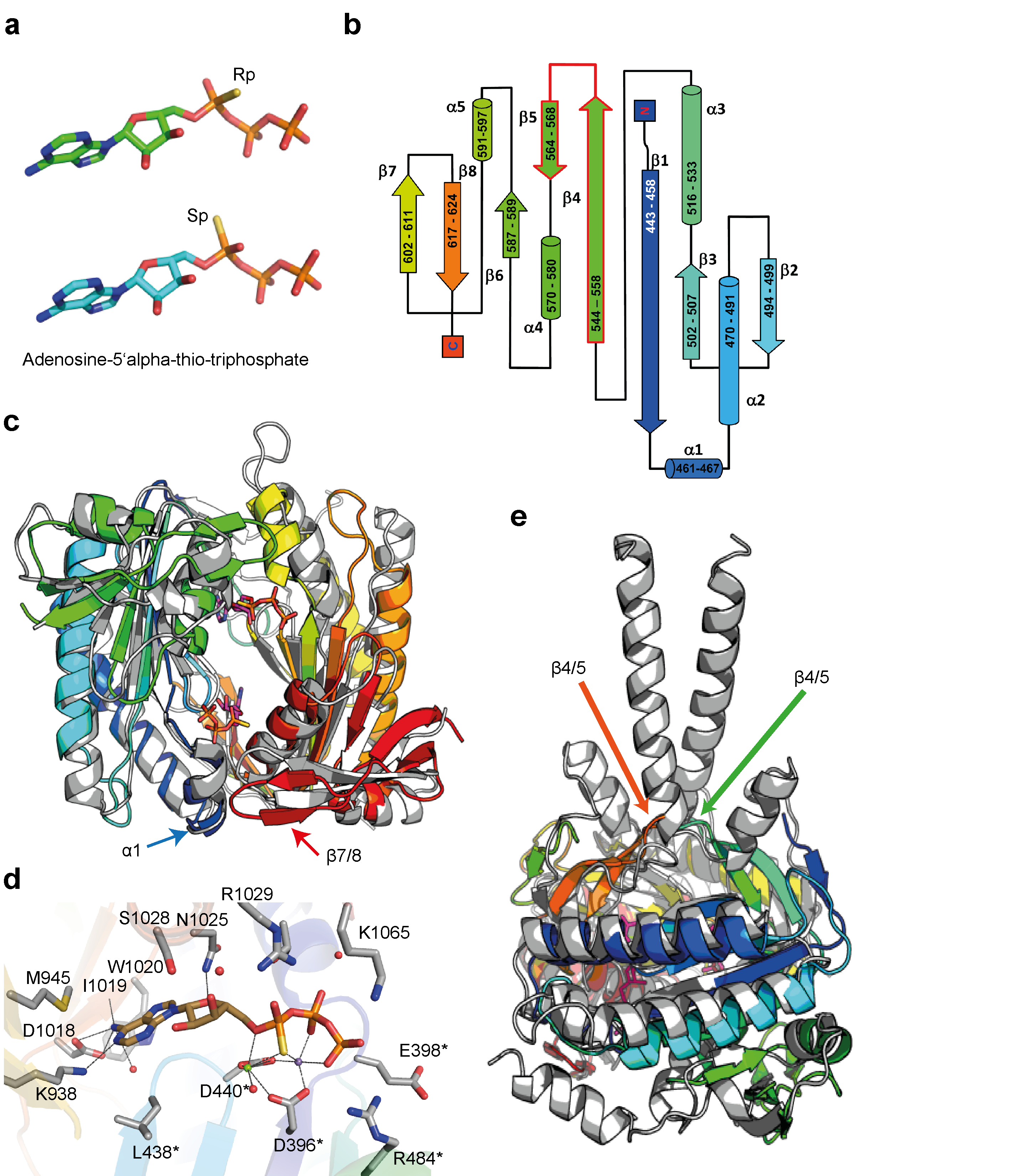


**Supplementary Fig 12 | Topology and structural comparisons of the double mutated adenylyl cyclases of *Catenaria* CaAC (E497K/C566D).** (**a**) Adenosine-5‘alpha-thio-triphosphate diastereomers Rp (top) and Sp (bottom). (**b**) Secondary structure elements according to Zhang et al^6^. (**c**) Overlay of CaAC (colored) with complexed tmAC (1CJK, gray), RMSD = 2.535 Å (311 to 311 atoms). Arrows indicate the protein closure, mediated by the approximation of α1 and β7/8 loop. (**d**) Active site CloseUP of the mammalian transmembrane adenylyl cyclase tmAC complexed with ATP-Rp-αS, Mg^2+^ and Mn^2+^ (1CJK). (**e**) Superimposition of the photoactivated adenylyl cyclase bPAC (5MBE, gray) and CaAC (structural alignment of cyclase domains: RMSD = 3.165 Å (229 to 229 atoms). Arrows indicate the clash between β4/5 of CaAC and the coiled-coil of bPAC. RMSD = root-mean-square deviation, grey lines indicate hydrogen bonds or metal coordination between 2.0 Å - 3.6 Å in length.


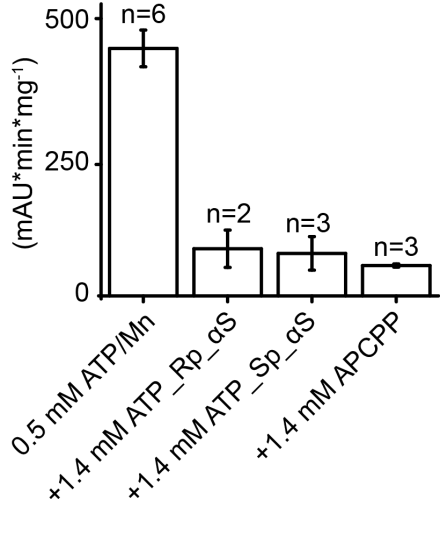


**Supplementary Fig 13 | The isolated adenylyl cyclase domain CaAC (E497K/C566D) was inhibited by ATP substrate analogs.** Purified CaAC(E497K/C566D) was incubated with 1.4 mM ATP-Rp-αS/ATP-Sp-αS/APCPP in the presence of 0.5 mM ATP/Mn^2+^ (pH = 7.5, incubation for 10 min). cAMP peak areas (mAU*min) per mg protein were quantified by RP-HPLC. CaAC was similarly inhibited by both ATPαS diasteromers. Bar graphs show data as means ± s.e.m.

**
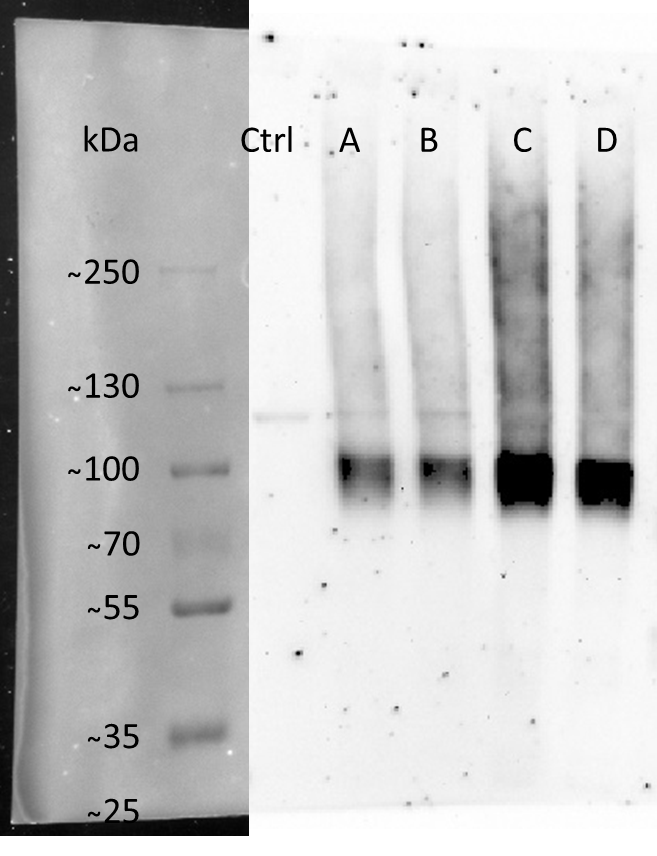
**

**Supplementary Figure 14 | The presence of a C-terminal YFP does not result in cleavage of BeRhGC-YFP expressed in *Xenopus* oocytes.** Immunoblot of oocyte lysates. Proteins were revealed using an anti-GFP antibody. In all samples, a single band was revealed at around 95 kDa corresponding to the full-length protein. Ctrl: non-injected oocyte, A: non-centrifuged homogenate from a single oocyte expressing BeRhGC-YFP, B: centrifuged homogenate (5 min, 12000g) from a single oocyte expressing BeRhGC-YFP, C: non-centrifuged homogenate from 3 oocytes expressing BeRhGC-YFP, D: centrifuged homogenate (5 min, 12000g) from 3 oocytes expressing BeRhGC-YFP.


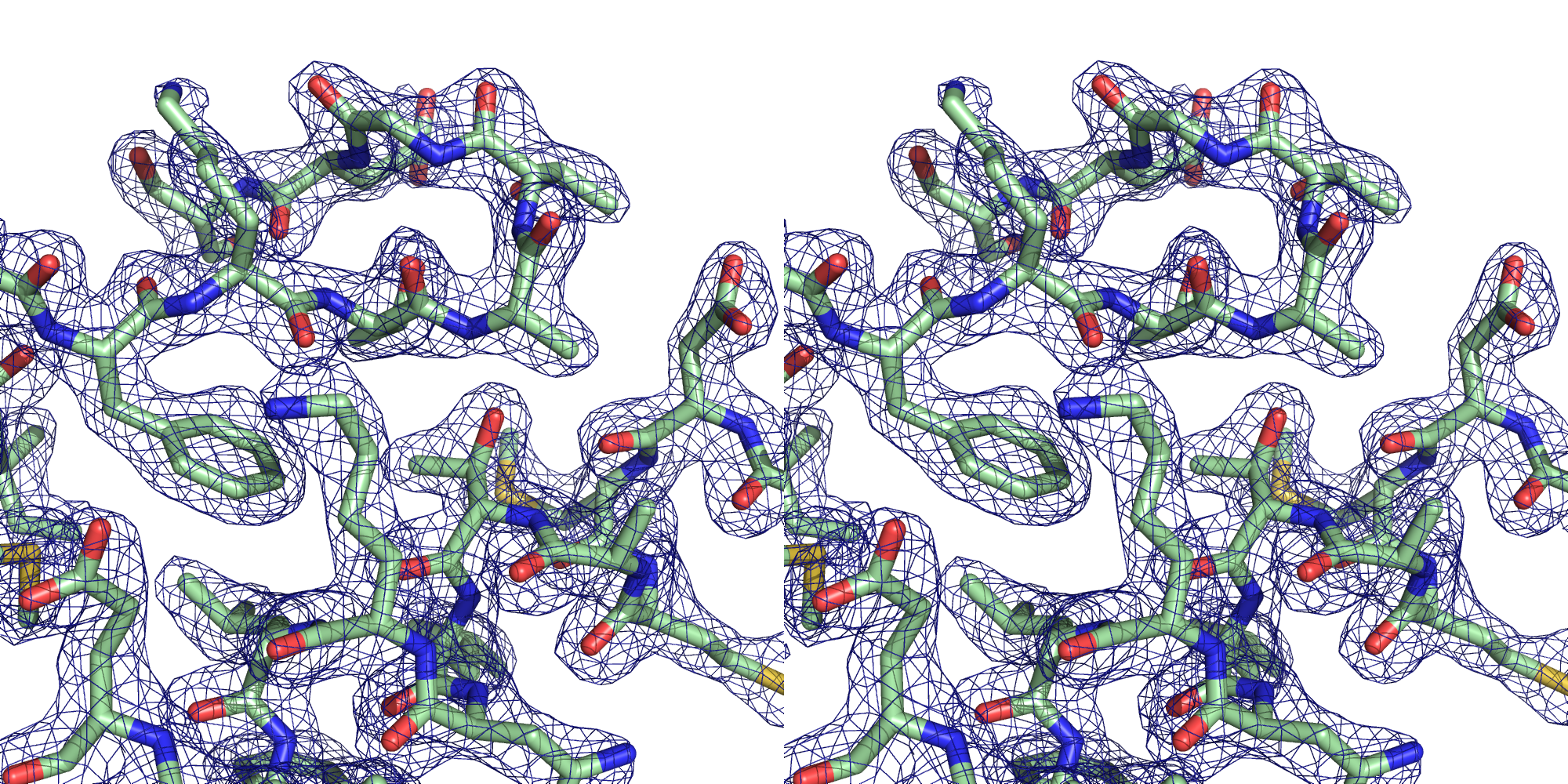


**Supplementary Figure 15 | Stereo image of representative electron-density for CaAC.**

Blue mesh shows the 2mFo-DFc map contoured at 1.5 sigma level, the crystallographic model is drawn as sticks (helix α 2 /3)**.**

**Supplementary Table 1 | Coiled-coil probability within the N-terminus of CaRhGC.** Prediction was done with COILS version 2.1^7^. Table shows values of window 14, probability of window 21 and 28 was <0.004. Compared to BeRhGC (Supplementary Table 3) coiled-coil probability is higher.

| Residue # | Residue |  | score | probability |
| --- | --- | --- | --- | --- |
| 117 | P | b | 1.25 | 0.01 |
| 118 | S | c | 1.50 | 0.19 |
| 119 | K | d | 1.56 | 0.33 |
| 120 | Q | e | 1.66 | 0.63 |
| 121 | S | f | 1.66 | 0.63 |
| 122 | A | g | 1.66 | 0.63 |
| 123 | L | a | 1.66 | 0.63 |
| 124 | Q | b | 1.66 | 0.63 |
| 125 | Q | c | 1.66 | 0.63 |
| 126 | Y | d | 1.66 | 0.63 |
| 127 | Q | e | 1.66 | 0.63 |
| 128 | T | f | 1.66 | 0.63 |
| 129 | N | g | 1.66 | 0.63 |
| 130 | I | a | 1.66 | 0.63 |
| 131 | A | b | 1.66 | 0.63 |
| 132 | D | c | 1.66 | 0.63 |
| 133 | M | d | 1.66 | 0.63 |

**Supplementary Table 2 | Coiled-coil probability within the N-terminus of BeRhGC.** Prediction was done with COILS version 2.1^7^. Table shows values of window 14, probability of window 21 and 28 was <0.004. Compared to CaRhGC (Supplementary Table 2) coiled-coil probability is low.

| Residue # | Residue |  | score | probability |
| --- | --- | --- | --- | --- |
| 73 | T | a | 1.08 | 0.00 |
| 74 | V | b | 1.26 | 0.01 |
| 75 | G | c | 1.36 | 0.04 |
| 76 | L | d | 1.36 | 0.04 |
| 77 | R | e | 1.36 | 0.04 |
| 78 | S | f | 1.36 | 0.04 |
| 79 | R | g | 1.36 | 0.04 |
| 80 | K | a | 1.36 | 0.04 |
| 81 | S | b | 1.36 | 0.04 |
| 82 | E | c | 1.36 | 0.04 |
| 83 | S | d | 1.36 | 0.04 |
| 84 | Q | e | 1.36 | 0.04 |
| 85 | A | f | 1.36 | 0.04 |
| 86 | N | g | 1.36 | 0.04 |
| 87 | V | a | 1.36 | 0.04 |
| 88 | R | b | 1.36 | 0.04 |
| 89 | G | c | 1.36 | 0.04 |
|  |  |  |  |  |
| 120 | G | e | 1.33 | 0.03 |
| 121 | S | f | 1.33 | 0.03 |
| 122 | A | g | 1.33 | 0.03 |
| 123 | L | a | 1.33 | 0.03 |
| 124 | A | b | 1.33 | 0.03 |
| 125 | N | c | 1.33 | 0.03 |
| 126 | Y | d | 1.33 | 0.03 |
| 127 | Q | e | 1.33 | 0.03 |
| 128 | S | f | 1.33 | 0.03 |
| 129 | A | g | 1.33 | 0.03 |
| 130 | M | a | 1.33 | 0.03 |
| 131 | S | b | 1.33 | 0.03 |
| 132 | E | c | 1.33 | 0.03 |
| 133 | L | d | 1.33 | 0.03 |

**Supplementary Table 3 | Summary of constructs used for experiments depicted in Figures 2, 6, Suppl. Fig. 3, 9-11.** As no photocurrents were evoked in some cells the total number of neurons recorded is also given, together with the percentage of responding neurons. All constructs used humanized codons except those designated as mouse. Data shown as median for I_Peak_, I_Sustained_ and Slope and as mean for the Cell parameters.

| Construct  Electrop. conc. | CNG channel  25 ng µl^-1^ | I_Peak_  (-pA) | I_Sustained_  (-pA) | Slope  (–pA ms^-1^) | Responding/  total cells (%) | Days of expression | Cell parameters | | |
| --- | --- | --- | --- | --- | --- | --- | --- | --- | --- |
|  |  |  |  |  |  |  | R_s_ | R_m_ | I_hold_ |
| BeRhGC  10 ng µl^-1^ | cGMP | 574 | 112 | 2.2 | 17/24 (70.8%) | 6-15 | 10.8 | 142.0 | 12.5 |
|  | cAMP | 35 |  |  |  | 14-15 | 8.4 | 241.2 | 9.5 |
| CaRhGC  10 ng µl^-1^ | cGMP | 1,260 | 236 | 7.2 | 16/44 (29.6%) | 6-8 | 10.7 | 128.3 | 15.2 |
|  | cAMP | 24 |  |  |  | 6 | 7.6 | 105.7 | 74.0 |
| YFP-CaRhGC (mouse)  10 ng µl^-1^ | cGMP | 787 | 251 | 8.7 | 18/20 (90%) | 10-11 | 7.3 | 112.8 | 5.2 |
|  | cAMP | 22 |  |  |  | 9 | 6.4 | 87.1 | -11.1 |
| CaRhGC-mycHis  100 ng µl^-1^ | cGMP | 2,049 | 388 | 28 | 12/15 (80.0%) | 8-9 | 13.8 | 167.5 | -22.1 |
|  | cAMP | 11 |  |  |  | 6-9 | 15.5 | 180.5 | 17.6 |
| YFP-CaRhGC-mycHis  100 ng µl^-1^ | cGMP | 342 | 73 | 1.4 | 7/15 (46.7%) | 7-8 | 6.1 | 86.6 | -14.9 |
| YFP-CaRhGC  10 ng µl^-1^ | cGMP | 1,441 | 146 | 7.7 | 14/16 (87.5%) | 9 | 7.3 | 86.8 | 36.8 |
| BeRhAC  25 ng µl^-1^ | cAMP | 617 | 591 | 0.8 | 11/14 (78.6%) | 6-8 | 12.4 | 132.4 | -94.6 |
|  | cGMP | 33 |  |  |  | 8 | 10.3 | 135.6 | -23.0 |
| CaRhAC  100 ng µl^-1^ | cAMP | 691 | 544 | 2 | 10/15 (66.7%) | 6-7 | 12.8 | 123.5 | -39.0 |
|  | cGMP | 23 |  |  |  | 7 | 10.1 | 110.5 | 1.7 |
| YFP-CaRhAC (mouse)  25 ng µl^-1^ | cAMP | 693 | 667 | 0.7 | 11/11(100%) | 8 | 8.6 | 117.1 | -66.5 |
|  | cGMP | 85 |  |  |  | 7 | 10.1 | 95.0 | -17.3 |
|  | - | 29 |  |  |  | 7 | 7.8 | 139.4 | -54.5 |
| CaRhAC-mycHis  100 ng µl^-1^ | cAMP | 536 | 523 | 1.1 | 12/30 (40.0%) | 6-8 | 9.5 | 151.6 | -29.7 |
|  | cGMP | 23 |  |  |  | 9-12 | 9.6 | 148.5 | -4.6 |

**Supplementary Table 4 | Overview of the constructs used**

| # | Cyclase name | experiment | N’ | YFP | linker | RhGC/  cyclase | linker | Overhangs & tags |
| --- | --- | --- | --- | --- | --- | --- | --- | --- |
| 1 | YFP^C^-CaRh-YFP^N^  (mouse) | BifC in oocytes | MAD | KQK...LYK (YFP^c^) | SR FCYENEVGTD | KD...AIL | EK SRITSEGEYIPLDQIDINVVDTS SR | MVS...IMA(YFP^N^) |
| 2 | CaRhGC-mychis | TEVC oocytes | MS |  |  | MKD...ARK | SY | Myc-his:  EQKLISEEDLKGSHHHHHH |
| 3 | CaRhGC | ELISA | MKD |  |  | MKD...ARK |  |  |
| 4 | CaRhGC-YFP  (mouse) | ELISA |  |  |  | MKD...ARK | LEK SRITSEGEYIPLDQIDINVVDTS SR | MVS...LYK SR FCYENEV |
| 5 | YFP-BeRhAC  (mouse) | ELISA | MVS | KGE...LYK | LEK | KD...GRK |  |  |
| 6 | YFP-BeRhAC-6x  (mouse) | ELISA | MVS | KGE...LYK | LEK | KD...GRK |  |  |
| 7 | YFP-CaRhAC-6x  (mouse) | ELISA | MVS | KGE...LYK | LEK | KD...ARK |  |  |
| 8 | BeRhAC | neuron patch-clamp |  |  |  | MKD...ARK | SVPAA | T2A:  EGRGSLLTCGDVEENPG |
| 9 | BeRhGC | neuron patch-clamp | MS |  |  | MKD...GRK | SVPAA | T2A:  EGRGSLLTCGDVEENPG |
| 10 | CaRhAC | neuron patch-clamp | MSMS |  |  | MKD...ARK | SVPAA | T2A:  EGRGSLLTCGDVEENPG |
| 11 | CaRhGC | neuron patch-clamp | MSMS |  |  | MKD...ARK | SVPAA | T2A:  EGRGSLLTCGDVEENPG |
| 12 | YFP-CaRhAC (mouse) | ELISA/neuron patch-clamp | MVS | KGE...LYK | LEK | KD...ARK |  |  |
| 13 | YFP-CaRhGC (mouse) | neuron patch-clamp | MVS | KGE...LYK | LEK | KD...ARK |  |  |
| 14 | CaRhAC-mycHis | neuron patch-clamp | PELMS |  |  | MKD...ARK | SY | Myc-his:  EQKLISEEDLKGSHHHHHH |
| 15 | CaRhGC-mycHis | neuron patch-clamp | PELMS |  |  | MKD...ARK | SY | Myc-his:  EQKLISEEDLKGSHHHHHH |
| 16 | YFP-CaRhGC-mycHis | neuron patch-clamp | MVS | KGE…LYK | ELMS | MKD…ARK | SY | Myc-his:  EQKLISEEDLKGSHHHHHH |
| 17 | YFP-CaRhGC | neuron patch-clamp | MVS | KGE…LYK | ELMS | MKD…ARK |  |  |
| 18 | CaRh | Expression in insect cells, spectroscopy |  |  |  | MKD...STV |  | TEV-his:  ENLYFQGHHHHHHHH |
| 19 | CaAC | Expression in *E.coli*/  Kinetics, xtal |  |  |  | MTEA…ARK | LE | His: HHHHHH |
| 20 | CaGC | Expression in *E.coli*, kinetics |  |  |  | MTEA...ARK | LE | His: HHHHHH |
| 21 | BeGC | Expression in *E.coli*, kinetics |  |  |  | MMTEA…GRK | LE | His: HHHHHH |
| 22 | CaRhGC | Expression in insect cells,  kinetics |  |  |  | MKD...ARK |  | TEV-his:  ENLYFQGHHHHHHHH |
| 23 | CaRhAC | Expression in insect cells,  Activity tests |  |  |  | MKD...ARK |  | TEV-his:  ENLYFQGHHHHHHHH |

**References**

1. Avelar, G. M. *et al.* A rhodopsin-guanylyl cyclase gene fusion functions in visual perception in a fungus. *Curr. Biol.* **24,** 1234–40 (2014).

2. Drozdetskiy, A., Cole, C., Procter, J. & Barton, G. J. JPred4: a protein secondary structure prediction server. *Nucleic Acids Res.* **43,** W389–W394 (2015).

3. Scheib, U. *et al.* The rhodopsin-guanylyl cyclase of the aquatic fungus Blastocladiella emersonii enables fast optical control of cGMP signaling. *Sci. Signal.* **8,** rs8 (2015).

4. Sievers, F. *et al.* Fast, scalable generation of high-quality protein multiple sequence alignments using Clustal Omega. *Mol. Syst. Biol.* **7,** 539 (2011).

5. Linder, J. U. & Schultz, J. E. The class III adenylyl cyclases: multi-purpose signalling modules. *Cell. Signal.* **15,** 1081–9 (2003).

6. Zhang, G., Liu, Y., Ruoho, A. E. & Hurley, J. H. Structure of the adenylyl cyclase catalytic core. *Nature* **386,** 247–53 (1997).

7. Lupas, A., Dyke, M. Van & Stock, J. Predicting coiled coils from protein sequences. *Science (80-. ).* **252,** 1162–1164 (1991).
